# On Infanticide

**Published:** 1820-03

**Authors:** W. Hutchinson

**Affiliations:** Sackville-street


THE LONDON

### Medical and Physical Journal.

3 OF VOL. XLIII.] MARCH, 1820. [NO. 253.

"For many fortunate discoveries in medicine, and for the detection of numerous "errors, the world is indebted to the rapid circulation of Monthly Journals; "and there never existed any work to which the Faculty in EUROPE and "AMERICA were under deeper obligations than to the Medical and Physical "Journal of London, now forming a long, but an invaluable, series." RUSH.

### Original Communications, Select Observations, etc.

### MEDICAL JURISPRUDENCE.

### DISSERTATION II.—*On Infanticide.*

### § I. *On the Physiological Relations of Infanticide.*

BY *infanticide*, in relation to jurisprudence, is meant the wilful destruction of the life of an infant within a short period, that is a few weeks, from the time of its birth. It is thus distinguished from *homicide*, because it is here considered that infanticide may be effected by intentional *omission* of proper care, as well as by *commission* of destructive violence.

Let us suppose that the body of a newly-born infant, apparently dead, is placed in the presence of a medical practitioner, and his decisions required respecting it, in relation to medical jurisprudence: the following should be his mode of conduct on this occasion.

He will first determine, as exactly as possible, whether there are any remains of life in it; and then make enquiries respecting the situation in which it was found, whether in a retired or hidden place, or if it had been from its birth constantly in the presence of known persons. If the latter be the case, whether or not it had shown signs of life; and, if it had evinced them, what were the circumstances relating to it previous to its death, (supposing it to be dead;) especially if it had cried, taken food, and voided its urine and fæces; and the length of time it has been dead. He will enquire respecting the mother, if known, whether this was her first child; if her labour had been natural, easy, or difficult, and if it had presented any extraordinary circumstances; what was the state of her health before and after parturition, especially whether she had suffered hæmorrhage; if any other persons were present at her delivery, and what are their social relations to her. If the body has been discovered in a retired place, he will ascertain the nature of the place; especially whether in water or on land; cold or hot; and if there were any circumstances about it calculated to accelerate or to retard the putrefaction of a dead animal body; whether the infant was clothed, and in what manner; especially if it were merely enveloped in some common garment, or dressed in the customary way of newly-born children. Its sex should also be noticed. Several of these are *moral* relations; but the knowledge of them may furnish useful indications for the mode of prosecuting this part of the enquiry. The next subjects for investigation are, whether the placenta remains attached to the infant; the length of time it appears to have been dead;* and (having washed the body, if necessary) if there are any signs of putrefaction in it, and the degree of them.

The grade of maturity of the infant, with respect to intra-uterine life, should then be determined, as exactly as possible;† especially whether it be nearly or quite complete,‡ and therefore furnish presumptive evidence of the capability of the subject to maintain life as an infant, provided there does not exist in its natural conformation any physical obstacle to its vitality.

The circumstances he has to keep in view in the further prosecution of his examination, are, 1°, Whether the infant was born dead or living, and if it lived after its birth; 2°, if it was born dead; whether it died some time before, or during, parturition; 3°, if it is proved to have lived after its birth; whether or not its death should be imputed to wilful destructive violence, or to omission of proper care; 4°, in case of its death having apparently arisen from want of due care; whether or not the mother was able to afford it.

The next point for particular investigation is, whether there are, externally, any clear and decisive marks of destructive violence on the infant; and, in case of the absence of them, it will be necessary to examine the body with great care and minuteness, especially about the head, (having shaved it, if the scalp be hairy,) eyes, nostrils, ears, mouth, anus, and vagina if it be a female. Many instances have occurred of infants having been destroyed by the introduction of a long needle, or similar instrument, through some of the parts above designated. Guy-

* There will be additional means for effecting this developed in the course of his examination; but he should now mark the external appearances.

† For the means for effecting this, see the dissertation on *Feticide*, pages 4-8 of the 251st Number of this Journal.

‡ This is a very important circumstance in relation to *social compacts* in some nations, where a fetus may be born alive, and continue to live for a few minutes or hours, and yet not be considered as an infant in some legal views, because it is not sufficiently mature to possess *vitability as an infant*, according to the common course of nature. Hence arose the serious disputations on the *vitability of the fetus* which appeared in the last two centuries. In England, if a fetus live after its birth, it is to all intents and purposes a *living child*. This point will be treated in a particular manner on a future occasion.

PATIN relates, too, that a *midwife* was hung at Paris for having made a practice of thrusting such an instrument into the head by one of the fontanels, as it presented in the vagina during its birth. In such cases, there will be a spot of ecchymosis, which will point out the wound; and this should be very carefully traced throughout its whole extent, with as little disturbance as possible of parts not immediately connected with it. Cases are recorded in which the brain and the heart have been lacerated, and their texture destroyed to a great extent, by such means.* The cavities of the mouth and nostrils should be examined, to ascertain also, whether they are, or appear to have been, artificially obstructed; especially if the infant has been found plunged into mud. Persons have first suffocated children by filling their mouth and nostrils with mud, and then placed them in it, supposing they could thus deceive the medical enquirer.

When ecchymosis and wounds are present, great care must be taken in order to determine whether they have arisen from natural circumstances attending parturition, or have been artificially effected; and, if the latter be the case, whether they were produced during life or after death.

With respect to ecchymosis, it should be borne in mind, that if the mother be a young woman, formed in the ordinary manner, and suffering her first parturition, when the infant is of the common size, and it has presented with the occiput inclined a little obliquely forwards and towards the left side of the mother, which is the ordinary and most favourable position, a tumefaction of the part thus engaged may generally be observed, the extent of which varies in different cases. If this tumor be dissected by the knife, more or less serous effusion is found, and a congestion of blood in the vessels, to an extent which does not exist in other parts of the head. When the labour has been long and difficult, especially if the head be unusually large, and has been pressed for a considerable time against the mouth of the uterus, when not fully dilated, by violent uterine contractions, a tumor of considerable size will often be formed, and sanguineous serum effused in the adjacent cellular tissue: sometimes the pericranium is even detached from the skull, and coagula of blood are found beneath it. The membranes uniting the sutures in this part may be elongated, and even lacerated; and consequently, the bones thus united, will be more easily and freely movable than in the ordinary state. When there is deformity of the pelvis of the mother, especially if it consist in a projection forward of the lower part of the lumbar vertebræ,

* There can be but little or no difficulty in determining, in the generality of cases, whether or not such violence was itself calculated to destroy life. The particular consideration of this subject must be deferred until *homicide by wounds* is treated.

there may exist, as a consequence of it, depression, and even fracture, of some one or more of the bones of the skull, which are commonly the parietal bones. When the breach has presented, there may be similar appearances of the skin and cellular tissue in this part; whilst the scalp, on dissection, shows spots of ecchymosis of a reddish colour dispersed about it, either above or beneath the pericranium. This is commonly found where infants have been turned, and delivered by the feet.* It cannot be difficult to distinguish those lesions from wilful destructive violence, if the situation of them is considered, and their degree: besides this, natural injuries of this kind, if destructive of life, would prevent the establishment of perfect respiration. This point will presently be considered in a particular manner.

A woman suffering labour alone may have the fetus escape from her, and fall to the ground on its head, whilst she is resting on her knees and elbows, or standing on her feet. Some experiments were therefore made at the *Hospice de la Maternité* at Paris, to ascertain what the effects on the head of the infant would generally be from such accidents. LECIEUX relates that fifteen infants were chosen who died a short time after their birth, in whom no deviation from the ordinary state in the bones of the skull could be discerned. They were raised up by the feet to such a height, that the surface of the sinciput was about twenty inches (English measure) from the ground, when they were let fall perpendicularly on a stone-floor. A longitudinal or angular fracture of one, and in some instances of both, parietal bones, was found in twelve of them. Fifteen infants were let fall in the same manner from twice the height above mentioned; and dissection showed in twelve of them fracture of the parietal bones, which in some extended to the os frontis. When an infant was let fall from a greater height, the membranous commissures of the cranium were stretched, and even torn in some places; the form of the brain often altered; and in some cases there was extravasation of blood above or beneath the dura mater, or in that membrane itself; and it was only when the cranium was unusually soft and flexible, that fracture was not found.

Contusions effected during life may be easily distinguished from those made after death, and from the common lividness of dead bodies. There is, in the first, always some degree of elevation of the skin; generally laceration of it; and there is constantly extravasation of blood. The parts where they are present will furnish some useful information respecting their

---

* LECIEUX, *Considerations sur l'Infanticide*, p. 25; HESSELBACH, *Vollst. anl. zu Gesetzmassigen Leichenoffn*; BUTTNER, *vom Kindermord*, p. 152.

origin. Ecchymoses from external violence during life are particularly evident in the course of the muscles over the bones; and, when wilfully effected, they generally correspond to some vital organ. Ecchymoses occurring after death are on a level with the surrounding skin, and ordinarily do not affect the *skin itself.* Lividity from tendency to putrefaction may be readily discerned by other co-existent circumstances.

Wounds made during life may be distinguished from those happening after death, with great facility; that is, before decomposition of the body has begun to take place. The former are red, sanguineous, and have their edges somewhat tumid and livid; there will be also extravasation of blood in the adjacent cellular tissue, and some degree of cutaneous ecchymosis. The latter are somewhat pale; their lips or edges are level with the adjacent skin, whitish, and flaccid. Their extent and situation will also furnish important indications respecting their origin.

The state of the spinal column should be particularly examined, especially about the neck, with a view to ascertain if there be luxation of any of the vertebræ, or marks of twisting or other analogous violence about them. If any luxation be present, care should be taken to examine whether there are such marks of violence on the skin as indicate great pressure on it during life: those are, excoriation, ecchymosis with tumefaction, and foldings or puckerings of it. Wounds about this part should be carefully traced, to see if they enter the spinal marrow.

The next enquiry will be, whether there are signs on the body externally of means capable of producing suffocation; because asphyxia caused by the viscid mucus naturally existing about the pharynx and glottis in newly-born infants getting into the trachea, *especially if the infant has lain on its back for some time after its delivery,** convulsive cough, and irregular action of the respiratory organs, may present internally similar appearances to those observed after suffocation from external violence: and therefore HEBENSTREIT correctly remarks,† that, unless signs on the body externally concur with those, it will hardly be possible, in any instance, to affirm that suffocation has been wilfully produced.

The livid circle round the neck, which, without due consideration, might seem to be a proof of the application of criminal violence, will, on proper reflection, appear to be capable of being produced by rigid and forcible contraction of the orifice of the uterus, or from the navel-string being twisted round the neck of the infant, which may have suffocated it.

---

* BUTTNER, *vom Kindermord*, p. 197.

† *Antropologia Forensis*, p. 431.

It is proved that an infant may respire whilst in the uterus, when its mouth presents to the dilated orifice of that organ. Some respectable men have denied the possibility of this,* which it is very easy to do; but other at least equally judicious men and well-informed physiologists, have thought it possible;† and several obstetric practitioners of great respectability and worthy of credit, have stated that they have heard the infant cry whilst in the uterus, in the situation above mentioned.‡ It must be sufficiently evident to every medical practitioner, that the infant may perish during its birth after respiration has thus taken place. It is possible that the navel-string may be twisted round the neck of the infant, but loosely until the body is nearly expelled, and then, if the placenta be firmly retained in the uterus, it may become tightened, and cause suffocation. These circumstances are possible when there is no person about the woman to render her proper assistance; and therefore, careful examination is necessary, in order to ascertain if, with the livid circle round the neck, there are marks of nails or points of fingers, or excoriation of the skin. The breadth of the mark, too, and whether or not it makes a complete circle, with the ends exactly meeting, and without deviating from this circle, should be noticed: the latter circumstances conjoined cannot arise from a natural twisting of the navel-string. The livid part should be carefully dissected, in order to ascertain if there are ruptured blood-vessels corresponding to it, if the trachea or larynx be flattened, or the cartilaginous rings of them laterally compressed; because these things indicate wilful violence, as such great injury does not take place from the natural twisting of the navel-string. Plouquet remarks on this point,§ that the signs of suffocation in doubtful cases are not only to be individually and collectively regarded, but the *degree* of them must also be considered, if we would draw any decisive evidence from them. In the cases of respiration before

---

* Amongst whom may be expressly designated Camper (in his *Eene Grechtelyke en Onleedkundundige Verhandeling over de tekenen van leven en dood in de nieuwgeboorene kinderen*), and Roederer (*Satura de Suffocatis.*)

† As Morgagni (*De Sedibus et Causis Morbor.* t. i. p. 188); Haller (*Element. Physiol.* t. viii. p. 400); Hunter (*On the Uncertainty of the Signs of Murder in the Case of Bastard Children*); Plouquet (*Comment sur Homicid.* p. 248); Techmeyer (*Med. Leg.* p. 243); Roux (*sur les Pertes de Sang, &c.*); Kannegiesserq (*Institut. Med. Leg.* p. 187); and Franck (*Program. Puerper. de Infant. susp. Opus. Med.* t. xii. p. 204.)

‡ These are, Idena (*Gedagten om het Dryven en Zinken der Longe Leeuward*); Croezer (*Ontwerp van de eerste Inademing*); Ficker (*Beytraege zur Arzneym,* Heft. ii. p. 130); Schmitt (*Neue Vers. und Erf. über die Lungenprobe*); Ossiander (Salzburg, *Medizinische-Chirurgische Zeitung*, 1809, Band. ii. p. 27); Siebold (*Ann. der Entbindungskunst*, b. i. p. 100); and Thilenius (*Loder's Journal,* iv. p. 638.)

§ *Opera citata*, p. 333.

birth, above indicated, it is indubitably certain that but a very partial dilatation of the lungs can take place, and that the changes properly insisted on by Plouquet as necessary, in order to rest decisive evidence on this point, can have happened only in a very slight and partial manner, as will be hereafter shown. FODERE states,* that instances have been known of suffocation having been produced by turning back the tongue on the glottis. This, it seems certain, must be the effect of wilful violence: Foderé, however, asserts this only with the following modifications: "when the infant has not sucked, and when the frenum of the tongue is lacerated." No medical practitioner has related any instance of accidental death from this cause. A livid, swoln state of the face and head must not be supposed to indicate suffocation; because these appearances are particularly observable in those cases where the navel-string happens to gird the child's neck, and where its head has been born some time before the body.†

The next subject for examination is the state of the umbilical cord; as it is certain that death may result from hemorrhage from this part, when a ligature has not been applied to it. TEICHMEYER,‡ HEBENSTREIT,§ and HALLER,‖ supposed, that if the placenta remained attached to it, it would be fatal to the life of the infant; but PLOUQUET¶ only admits the truth of this opinion when the expulsion of the placenta has been simultaneous with that of the fetus, when sufficient hemorrhage may take place from it to destroy the life of the infant. Some few instances have occurred of a knot being formed in the cord; and such an accident may have been the cause of death, by its being drawn so tight as to stop the circulation in it before delivery of the fetus. Prolapsus of it may, as every medical practitioner must be aware of, be attended with the same result. The remarks made respecting the state of the infant, in the case of death occurring during birth, after imperfect respiration had been effected, may become applicable to this case also.

If the placenta be not attached to it, it should be observed whether the cord has been cut or torn, if there are marks of powerful twisting or ligature of it, and at what distance from the body the separation was made.

About the termination of the seventeenth century, doubts were first advanced respecting the necessity of tying the cord

---

* *Med. Legale*, tom. iv. p. 495.

† HUNTER, *on the Uncertainty of the Signs of Murder in the Case of Bastard Children.*

‡ *Opera citata*, p. 246.

§ *Antropolog. Foren.* p. 417.

‖ *Element. Physiol.* t. x.

¶ *Opera citata*, p. 331.

to prevent hemorrhage,* which gave rise to much disputation; and, although the error of the doctrine of the innovators soon became generally acknowledged, it is of considerable importance in medical jurisprudence that a knowledge of the facts developed chiefly by that disputation should be acquired, and their relation to the subject under consideration pointed out.

The ligature of the umbilical cord is not indispensably necessary in all cases, for under certain circumstances it may remain free, without the occurrence of hemorrhage; yet there are examples of fatal hemorrhage having resulted from it. Robust infants are more liable to hemorrhage, in the absence of the ligature, than those which are weak. The period at which the division of the cord was made, has much influence on the results of it: hemorrhage is much less to be feared when it has been effected after the infant has breathed and cried, (that is, when the new course of the circulation has become established,) than before this has taken place. The nearer the separation is to the body of the infant, the greater will be the danger of hemorrhage. When the cord has been cut by a sharp instrument, there is a greater disposition to bleeding from it than when it has been torn. It should be borne in mind, that the infant may be suffered to perish from hemorrhage from the navel-string, and then a ligature may have been applied to it: it will therefore be necessary to notice whether or not there are signs of death from such a cause, although the cord may be tied. Those signs are, a bluish or dull pallid colour of the whole surface of the body, paleness of the viscera, want of blood in the large vessels, especially the veins, and in the auricles of the heart. But such effects cannot be attributed to hemorrhage from the navel-string, except when no other means for its occurrence are present, and when the body of the infant appears to be well constituted, with a full and free development of the cord. When this part is shrivelled, and its vessels in a state of extreme collapse, it may be supposed that the infant wanted blood for some time before its birth. Although there may be evidences of umbilical hemorrhage, it must not therefore be concluded that

---

* By FANTON, professor at Turin, (*Anat. Human. Corp.* p. 251), and towards the middle of the last century, the dispute was warmly excited by ALBERTI, in 1731; and SCHULTZIUS, in a dissertation entitled *An umbilici deligatio in nuper natis absolute necessaria sit?* Halle, 1733; ELLER (*Com. Lit. Nov. an.* 1733, p. 377); TREVIUS (*Epist. Med. For. de Fetu, &c.*); SCHAEL (*De Funic. umb. del non absolut. necess.*); KALTSCHMIED (*De Intermissa Fun. umb. delig. non absolut. necess.*); SCHWEIKHARD (*De non necessar. funic. umb. deligat.*); and BURTON (*New System, &c.* p. 61,) agreed that it was not necessary: whilst BOEMER (*De necess. fun. umb. deligat.*); HEISTER (*Dissert. de sum. necees. inspect. cord. vasorumque major. sub. legali infant. sect.* § xiii.); HALLER (*Element. Physiol.* t. x. p. 200); and PLOUQUET (*opera citat.* p. 326), entered the arena in favour of the necessity of the ligature; and at length, as Haller states, *Eo redierunt etiam adversæ sententiæ Medici, ut funiculum ligare juberent.*

it has arisen from a wilful criminal action, because it might have taken place from the placenta during labour; or, as Mr. BURNS states, rupture of the vessels of the cord may have happened at that time, and fatal hemorrhage thence resulted.* The coincidence of vacuity of the blood-vessels, with a division of the cord, and perfect dilatation of the lungs, may be considered to furnish proofs of the continuation of life after birth, and very strong presumptive evidence of wilful and criminal negligence in the mother, (supposing her to have been delivered whilst alone;) as, if she were able to separate the placenta, it may be very properly considered that she was also able to apply a ligature to the cord; and the same intelligence that would lead her to do the former, must also lead her to effect the latter. But, the coincidence of signs of *imperfect respiration*, with vacuity of the blood-vessels in consequence of hemorrhage from the placenta or navel-string, cannot alone furnish evidence of the existence of life after birth. The presence of a considerable quantity of blood about the place where the infant has been found, or in the linen enveloping it, with want of ligature of the cord, must not be considered evidence of its perishing by hemorrhage, unless examination of the state of the heart and great blood-vessels indicates it; because artful women have left the infant in that state covered with their own blood, (that is, the mother's,) for the purpose of the imposition here indicated.

If the child has been found in water, it becomes a question whether it was put into it when dead or living.† Should the former appear to have been the case, the general course of enquiry here described must be pursued. An infant may be left to perish in a cold place. But, as the exposition of it in this way is itself considered as infanticide by *omission* of proper care, at least, it can hardly in any instance be a matter of importance to determine whether or not the infant perished from the influence of cold expressly. The peculiar signs of this will be, great stiffness of the limbs, congestion of blood in the interior parts of the body, probably extravasation of it in some of the cavities, and absence of it exteriorly. Whilst the body of an infant already dead, exposed to the same circumstances, will be more lax and pliable, except it be actually frozen.

Three questions must arise at this stage of the enquiry, if they have not necessarily presented themselves before this time: 1°. Are there signs of *wilful* or *intentional* violence on the body, calculated to destroy its life? 2°. Did the infant continue to live after its birth? 3°. If marks of violence exist, was this

---

* *Principles of Midwifery*, p. 130; ROEDERER makes a similar observation, *Element. Artis Obstetric.* § 389.

† This point will be treated in a particular manner on a future occasion, in the dissertation on *Homicide*.

violence inflicted during infantine life? Unless the latter be determined in the affirmative, it cannot be asserted that *infanticide* has been committed,* because that violence may have been effected on a dead child. Though, if it be proved that the infant lived after its birth, there is presumptive evidence that such violence caused its death; because it cannot be supposed that any person would wilfully effect it on a child after it had sensibly ceased to live; though it may be imagined that it might be committed on a still-born child, to prevent the chance of its survivance. However, the court of justice will require physiological proofs of *infanticide*, before the penalty of it is put in force. The signs by which the injuries in question may be known to have been inflicted during life, have been already designated: those of the existence of extra-uterine life must be now described.

Although the most decisive signs of extra-uterine life are those of the existence of perfect respiration, it will be right to take cognizance, in the first instance, of all those presented by the body externally, which either tend to prove or to deny it. The former are, sufficient maturity of the body; the absence of any apparent natural physical obstacles to the continuance of life; a well-nourished, fresh, and natural development and colour of the body; sufficient length, consistence, and healthy formation, of the umbilical cord; and, if there are wounds or ecchymoses, the signs attending them designated on a former occasion. The presumptive signs of death for some time before birth, are, the easy separation of the cuticle without tendency to putrefaction, but with a soft, sodden state of the skin and adjacent structure, and a shrivelled, soft, and tender state of the umbilical cord, (similar characters should be sought for in the placenta, if it be found with the infant,) are very forcible evidences that the fetus had remained in the uterus after its death, unless it has been found in water. A soft pultaceous state of the muscles, and a fluctuation in the cellular tissue, especially distinct about the head, may also be commonly discerned in this case. The fontanels are also often sunken, the sutures of the cranium are relaxed, and the bones of the skull very movable. If death had preceded parturition a very long period, there may be great progress towards putrid decomposition, which may not be very easily distinguishable from that occurring under some circumstances after birth. The state of the bladder and great intestines may be next examined; but the emptiness of these in a mature fetus is not a very forcible indication of life after birth, because their contents are

* The other view which may be taken of those points was considered in the dissertation on *Feticide*.

sometimes evacuated during parturition, especially when the breech has presented. The state of the brain and abdominal viscera should then be observed, and any remarkable appearances noticed; and lastly, that of the lungs and pulmonary arteries.

W. HUTCHINSON.

*Sackville-street; Feb. 8th,* 1820.

